# Kissing Balloon Technique for Angioplasty of Tibioperoneal Arteries Bifurcation Using Pedal Arterial Retrograde Revascularization

**DOI:** 10.1155/2018/9543250

**Published:** 2018-12-25

**Authors:** Ahmed Amro, Obadah Aqtash, Adee Elhamdani, Mehiar El-Hamdani

**Affiliations:** ^1^Department of Cardiovascular Services, Marshall University Joan C. Edwards School of Medicine, Huntington, WV, USA; ^2^Department of Internal Medicine, Marshall University Joan C. Edwards School of Medicine, Huntington, WV, USA

## Abstract

**Background:**

Kissing Balloon Technique using retrograde pedal approach together with anterograde common femoral artery (CFA) approach could be the treatment of choice in patients with diseased infrapopliteal artery bifurcation. We report seven cases where the KBT was utilized for the treatment of diseased infrapopliteal artery bifurcation using retrograde pedal access in conjunction with the conventional common femoral artery (CFA) access.

**Methods:**

We reviewed all seven cases that underwent KBT with the combination of pedal and common femoral access in a single-center study from 2014 to 2015 utilizing Rutherford classification severity index; all cases were deemed stage 3 (severe claudication) to stage 6 (severe ischemic ulcers or frank gangrene). With the exception of two cases, contralateral femoral access was obtained, with sheath sizes varying from 4 to 6 French for both CFA and pedal access. Ultrasound was utilized for ipsilateral pedal access in all seven cases.

**Results:**

Arterial revascularization was successfully achieved by the KBT in all patients without any complications. All patients achieved procedural success, which is defined as residual stenosis of less than 30% with no dissection or thrombosis and clinical success that is defined as resolution of symptoms (absence of intermittent claudication and healing of the ulcer) as well as improvement in the arterial brachial index (ABI). During follow-up, out of the seven cases, repeat angiogram was performed for one case, which showed patent arteries with no residual lesions.

**Conclusions:**

In patients with popliteal and tibioperoneal trunk bifurcation lesions, Kissing Balloon Technique using retrograde pedal access in conjunction with the conventional anterograde access appeared to be successful, safe, and effective technique with lower access site complications and shorter procedure time.

## 1. Background

Tibioperoneal occlusive disease is among the most medically and surgically challenging problems in vascular surgery [1]. A popular treatment modality is single balloon angioplasty, which may shift the plaque into an adjacent untreated artery causing thrombosis, dissection, or spasm. Kissing Balloon Technique has been shown to be effective in avoiding these complications [2]. Considering the advancement of ultrasound technology for pedal access nowadays and with retrograde pedal access being more popular and widely used among physicians who are involved in peripheral intervention, KBT using retrograde pedal approach together with anterograde common femoral artery (CFA) approach could be the treatment of choice in patients with diseased infrapopliteal artery bifurcation. We report seven cases where the KBT was utilized for the treatment of diseased infrapopliteal artery bifurcation using retrograde pedal access in conjunction with the conventional common femoral artery (CFA) access.

## 2. Methods

We reviewed all seven cases that underwent KBT with the combination of pedal and common femoral access in a single-center study from 2014 to 2015 utilizing Rutherford classification severity index. All cases were deemed stage 3 (severe claudication) to stage 6 (severe ischemic ulcers or frank gangrene). With the exception of two cases, contralateral femoral access was obtained with sheath sizes varying from 4 to 6 French for both CFA and pedal access. Ultrasound was utilized for ipsilateral pedal access in all seven cases.

### 2.1. Kissing Balloon Technique

One wire was pushed down from an anterograde femoral approach to one of the two tibial arteries below the bifurcation being treated (anterior tibial artery and tibioperoneal trunk or posterior tibial artery and peroneal artery). The other wire was then brought to above the stenosed artery from a retrograde pedal approach. The operator chose balloon diameter and lengths based on the extent of the lesion and vessel diameters. Noncompliant balloons were used and positioned with half of the length of the balloon in the branch artery and half in the main artery. Balloons then inflated simultaneously for 1 to 2 minutes with procedural success defined as residual stenosis of less than 30% with no dissection or thrombosis. Follow-up was performed 3 to 8 months after the kissing balloon procedure. Patient's demographics, vascular symptoms, disease characteristics, procedural success, and complications were collected (Tables 1 and 2).

## 3. Results

The seven patients in this case series had a mean ± standard deviation age of 66.1 ± 6.93. Comorbidities in the patients included diabetes in 4 (57%), coronary artery disease in 5 (71%), and chronic kidney disease in 1 (14%). Arterial revascularization was successfully achieved by the KBT in all patients without any complications. All patients achieved procedural success, which is defined as residual stenosis of less than 30% with no dissection or thrombosis and clinical success that is defined as resolution of symptoms (absence of intermittent claudication and healing of the ulcer) as well as improvement in the arterial brachial index (ABI) (Table 2). During follow-up, repeat angiogram was performed for one out of the seven cases and showed patent arteries with no residual lesions.

## 4. Discussion

Dr. Gruentzig first used the term kissing balloon in 1980 to describe percutaneous treatment of iliac bifurcation lesions and then a year later he applied the technique to coronary interventions. The KBT is a procedure designed for percutaneous transluminal angioplasty of arterial bifurcation lesions [3]. It is the first percutaneous technique specific for bifurcation lesions and remains a mainstay of coronary interventions. Because of their anatomy, using an anterograde single balloon angioplasty for popliteal and tibioperoneal trunk bifurcation lesions exposes the patient to the risk of side branch damage, which may worsen the present stenosis and in some cases may cause side branch occlusion. There are different suggested mechanisms explaining side branch damage, including plaque shift, refractory spasm, or dissection of the ostium. The use of KBT helps in avoiding these complications with more favorable outcomes and lower restenosis rate [4].

KBT method for treatment of infrapopliteal bifurcations using only anterograde CFA approach has been previously described and published by Dr. Gargiulo in 2008 as a case series of 8 limbs, where it was shown to be effective and safe [2]. In this case series, our patients were successfully treated by KBT using retrograde pedal access in conjunction with anterograde CFA access.

We believe that using retrograde pedal access in conjunction with anterograde CFA access allows the usage of smaller sheath sizes for successful KBT as one wire and balloon will be advanced from the top while the other wire and balloon will be advanced from the bottom. Regarding our seven cases, the sheath size varied from 4 to 6F for both CFA and pedal access. Using smaller sheath sizes is associated with lower rates of access site complications especially in those patients with known severe peripheral artery disease (PAD) and heavily calcified vessels. Accessing the CFA has long been the traditional approach for treating the diseased arteries as well as delivering therapeutic options. To perform the KBT through a single anterograde access, a relatively larger sheath size is typically utilized, usually 7-8F sheath, where a single catheter with a double balloon or two balloon catheters are then pushed down separately into the anterior and posterior tibial arteries.

We also believe that the addition of retrograde pedal approach for KBT in this complex diseased cohort of patients is extremely valuable in achieving quicker therapy and shorter procedure time by reducing the likelihood of entering collateral vessels while trying to cross the actual lesions as these collaterals arise at caudal angles [5, 6]. In addition, the success of pedal approach is likely influenced by better push ability for the wires, balloons, and devices due to relatively smaller artery diameter and close proximity of the chronic total occlusion (CTO) segment of the artery to the access site [7]. It is important to note that utilization of ultrasound is essential for pedal access since it improves accuracy and minimizes complications [6, 8]. In our case series, pedal access was achieved from the first attempt using ultrasound in all patients. The possibility of multiple sticks to gain access in the absence of ultrasound utilization may lead to vessel spasm, thrombosis, pseudoaneurysm development and AV fistula formation, or obliteration of the runoff, which leads to poor outcomes [7, 9].

## 5. Conclusion

In patients with popliteal and tibioperoneal trunk bifurcation lesions, Kissing Balloon Technique using retrograde pedal access in conjunction with the conventional anterograde CFA access could be the treatment of choice as it appeared to be successful, safe, and effective technique with lower access site complications and shorter procedure time.

## Figures and Tables

**Table 1:** Demographics, risk factors, clinical presentation, and Rutherford class at presentation.

**Patient number**	**Age**	**Gender**	**BMI**	**Risk factors**	**Symptoms**	**Rutherford**
**1**	52	M	33.9	Smoking DM CKD	Nonhealing ulcer	6

**2**	69	F	29.3	Smoking DM CAD	Intermittent claudication	3

**3**	67	F	28.6	None	Intermittent claudication	3

**4**	66	F	26.2	DM CAD	Intermittent claudication	3

**5**	67	F	35.9	Smoking CAD	Intermittent claudication	3

**6**	67	M	29.0	Smoking DM CAD	Intermittent claudication	3

**7**	75	M	39.5	Smoking CAD	Nonhealing ulcer	6

M, male, F, female, BMI, body mass index, DM, diabetes mellitus, CAD, coronary artery disease, CKD, chronic kidney disease.

**Table 2:** The arterial lesions, endovascular access, and follow-up for the 7 patients.

**Patient number**	**Limb**	**ABI**	**TBI**	**Arterial lesion site**	**Access site**	**Sheath**	**Follow-up**
**1**	Right	(0.41)	(0.33)	(i) Right PA 50-60% stenosis (ii) Right peroneal artery 50-60% stenosis (iii) Right anterior tibial artery completely occluded (iv) Right posterior tibial artery completely occluded	Right femoral artery Right anterior tibial artery	6 French sheath 4 French sheath	5 months' follow-up: total healing of ulcer with repeat ABI: 0.73.

**2**	Left	(0.85)	(0.5)	(i) Left anterior tibial artery 80% stenosis proximally (ii) Left tibioperoneal trunk 80-90% stenosis proximally and 70% distally (iii) Left posterior tibial artery 80% stenosis proximally (iv) Left deep peroneal 60-70% stenosis proximally	Right femoral artery Left posterior tibial artery	6 French sheath 4 French sheath	4 months' follow-up: improved claudication with repeat ABI 0.91.

**3**	Left	N/A	N/A	(i) Left tibioperoneal trunk 50% stenosis (ii) Left posterior tibial 60% stenosis proximally (iii) Left deep peroneal artery 60-80% stenosis	Right femoral artery Left posterior tibial artery	6 French sheath 4 French sheath	3 months' follow-up: improved claudication with widely patent left popliteal and tibioperoneal seen on repeat angiogram.

**4**	Right	N/A	N/A	(i) Right femoral-popliteal bypass stenosis proximally and distally (ii) Right common iliac 80-90% stenosis (iii) Left common iliac 70% stenosis	Left femoral artery Right posterior tibial artery	6 French sheath 4 French sheath	7 months' follow-up: improved claudication.

**5**	Left	(0.63)	(0.32)	(i) Left distal SFA and PA stenosis (ii) Left tibioperoneal trunk and posterior tibial stenosis (iii) Left deep peroneal artery stenosis	Right femoral artery Left deep peroneal artery	6 French sheath 4 French sheath	8 months' follow-up: improved claudication with repeat ABI 0.73.

**6**	Left	(0.49)	0.39)	(i) Left SFA 90-95% stenosis distally (ii) Left tibioperoneal trunk 70-80% (iii) Left anterior tibial artery completely occluded (iv) Left posterior tibial artery completely occluded	Right femoral artery Left anterior tibial artery	6 French sheath 4 French sheath	3 months' follow-up: improved claudication with repeat ABI 0.91.

**7**	Left	(0.59)	(0.44)	(i) Left popliteal artery 90% stenosis distally (ii) Left anterior tibial artery 70% stenosis distally (iii) Left tibial artery 80% stenosis (iv) Left peroneal artery occluded	Left femoral artery Left anterior tibial artery	6 French sheath 4 French sheath	5 months' follow-up: significant healing of ulcer with repeat ABI 0.66.

ABI, ankle brachial index, TBI, toe brachial index, N/A, not applicable, SFA, superficial femoral artery, PA, popliteal artery.
